# Accurate Strand-Specific Quantification of Viral RNA

**DOI:** 10.1371/journal.pone.0007468

**Published:** 2009-10-22

**Authors:** Nicole E. Plaskon, Zach N. Adelman, Kevin M. Myles

**Affiliations:** Fralin Life Science Institute, Department of Entomology, Virginia Tech, Blacksburg, Virginia, United States of America; Institut Pasteur Korea, Republic of Korea

## Abstract

The presence of full-length complements of viral genomic RNA is a hallmark of RNA virus replication within an infected cell. As such, methods for detecting and measuring specific strands of viral RNA in infected cells and tissues are important in the study of RNA viruses. Strand-specific quantitative real-time PCR (ssqPCR) assays are increasingly being used for this purpose, but the accuracy of these assays depends on the assumption that the amount of cDNA measured during the quantitative PCR (qPCR) step accurately reflects amounts of a specific viral RNA strand present in the RT reaction. To specifically test this assumption, we developed multiple ssqPCR assays for the positive-strand RNA virus o'nyong-nyong (ONNV) that were based upon the most prevalent ssqPCR assay design types in the literature. We then compared various parameters of the ONNV-specific assays. We found that an assay employing standard unmodified virus-specific primers failed to discern the difference between cDNAs generated from virus specific primers and those generated through false priming. Further, we were unable to accurately measure levels of ONNV (−) strand RNA with this assay when higher levels of cDNA generated from the (+) strand were present. Taken together, these results suggest that assays of this type do not accurately quantify levels of the anti-genomic strand present during RNA virus infectious cycles. However, an assay permitting the use of a tag-specific primer was able to distinguish cDNAs transcribed from ONNV (−) strand RNA from other cDNAs present, thus allowing accurate quantification of the anti-genomic strand. We also report the sensitivities of two different detection strategies and chemistries, SYBR® Green and DNA hydrolysis probes, used with our tagged ONNV-specific ssqPCR assays. Finally, we describe development, design and validation of ssqPCR assays for chikungunya virus (CHIKV), the recent cause of large outbreaks of disease in the Indian Ocean region.

## Introduction

Although the genomes of RNA viruses occur in a variety of conformations, all must be efficiently copied within the infected cell. These copies are essential to the production of messenger RNA (mRNA) that can be translated by host ribosomes, and as a source of genomic RNA for packaging into mature progeny virions. The alphaviruses are a group of enveloped, positive (+) strand RNA viruses in the family Togaviridae [1]. The synthesis of genomic (49S) RNA, as well as a subgenomic (26S) mRNA that encodes the virus structural proteins, depends on the synthesis of a genomic-length minus (−) strand copy. Alphaviruses are thought to synthesize (−) strand RNAs only for a short time early in the infection, although the production of (+) strand 26S and 49S RNA continues for much longer [2].

Members of the alphavirus genus pose a serious or potential threat to public health in many areas of the world. Nearly all alphaviruses are maintained in nature by transmission cycles that involve alternating replication in a susceptible vertebrate and invertebrate host. Because infection of the vertebrate host is acute and often associated with disease, continual transmission depends on life-long persistent infection of the invertebrate vector host, for many alphaviruses a mosquito. It is presently unclear how persistent alphavirus infections are maintained in the vector host, after (−) strand synthesis terminates in the infected cells. One of the difficulties in addressing this question has been the limitations of methodologies for detecting and measuring (−) strand RNA in infected cells. Competition between viral (+) strands and labeled probe makes nuclease protection assays problematic for the detection of (−) strand RNA, particularly late in the infection when (+) strands are much more abundant [3]. In addition, nuclease protection assays are only semi-quantitative. Assays based on reverse transcription (RT) and PCR of cDNA derived from viral (−) strands increase sensitivity at later times after infection, but are also semi-quantitative [4]. This weakness can be overcome with quantitative real-time PCR (qPCR), but the specificity of these assays for a particular strand of viral RNA is crucial to obtaining accurate and conclusive measurements.

Although a variety of strand-specific quantitative real-time PCR (ssqPCR) assays utilizing different designs, detection strategies and chemistries have been reported [5], [6], [7], [8], [9], [10], [11], [12], [13], [14], [15], [16], no study has yet determined if the specificity, accuracy and sensitivity of each is equivalent. Here we report on the development and validation of new ssqPCR assays for the alphaviruses o'nyong-nyong (ONNV) and chikungunya (CHIKV). Although the assays developed are specific for ONNV and CHIKV, different assay designs, detection strategies and chemistries were evaluated during the development process and those results are also reported here. We show that accurate quantification of a specific strand of viral RNA, in the presence of relatively higher levels of cDNAs generated from the complementary strand, requires incorporation of a unique tag sequence into cDNA generated during the RT step, and the use of a tag-specific primer during the qPCR step. Our results also indicate a greater dynamic range for tagged ssqPCR assays using DNA hydrolysis probes, when compared with those using SYBR® Green in the quantification of low copy templates. These findings should be useful in informing the design of future ssqPCR assays for the detection and accurate measurement of replicating viral RNA in infected cells and tissues.

## Materials and Methods

### Infecting mosquito cells with ONNV

*Aedes albopictus* C6/36 cell monolayers were grown to 80% confluency in 12-well plates, washed twice with PBS, and infected with ONNV at a multiplicity of infection (MOI) of 5. Virus was diluted with Dulbecco's Modified Eagle Medium (DMEM) (Mediatech, Inc.) to a total volume of 0.5 mL/well and placed on the cells at 4°C for one hour. After one hour, fresh medium was added to the wells bringing the total volume to 1 mL, and cells were placed at 28°C for 1 hour. Total RNA was isolated from ONNV-infected cells with TRI Reagent RT® (Molecular Research Center, Inc.) at 1 hour post infection.

### Generating in vitro RNA transcripts

To generate strand-specific standard curves for ssqPCR, (+) and (−) strand RNA was transcribed *in vitro* from a plasmid containing a portion of the nsP1 gene from either ONNV or CHIKV. The plasmids pblue-nsP1 (ONNV) and pblue-nsP1 (CHIKV) were produced by cloning the 5′ terminal 853 and 669 nucleotides of the respective viral nsP1 gene into pBluescript II SK (−) (Stratagene). Minus strand RNA was synthesized with T7 RNA polymerase from *Hind*III-digested plasmid templates in a standard in vitro transcription reaction. Positive strand RNA was synthesized with SP6 RNA polymerase from *Kpn*I-digested plasmid templates in a standard in vitro transcription reaction. The RNA generated during the in vitro transcription reactions was isolated with TRI Reagent RT®, as per manufacturer's instructions. The absence of template DNA was confirmed through PCR. The concentration of RNA transcripts was determined with a NanoDrop spectrophotometer (Thermo Scientific). The cloned ONNV nsP1 gene fragment has a molecular weight of 273,545 g/mol, while the cloned CHIKV nsP1 gene fragment has a molecular weight of 214,574 g/mol. One µg of RNA transcribed from pblue-nsP1 (ONNV) equals approximately 2.2×10^12^ molecules, while one µg of RNA transcribed from pblue-nsP1 (CHIKV) equals approximately 2.8×10^12^ molecules.

### Reverse transcriptase-PCR

cDNAs of both polarities were transcribed with primers containing a 5′ tag sequence [9], [17], or with primers lacking the 5′ tag sequence (Table 1). Forward primers were used to transcribe cDNA from (−) strand RNA, while reverse primers were used to transcribe cDNA from (+) strand RNA. Primers and RNA were incubated at 70°C for 5 min and then placed on ice for 2 min. Primer was added to the reverse transcription reaction at a final concentration of 500 nM. cDNA was synthesized with Superscript II® (Invitrogen) at 50°C for 30 min, and then heat inactivated at 95°C for 15 min. Unincorporated primers present in heat inactivated reverse transcription reactions were digested with exonuclease I (New England Biolabs). cDNAs used in the generation of standard curves were serially diluted (∼10^10^–10^2^ gene copies/µl) and stored at −20°C until further use.

**Table 1:** Sequence of primers and probes used for reverse transcription (RT) and quantitative PCR (qPCR).

Oligonucleotide Name	Purpose	Nucleotide sequence (5′→3′)a,b
**SYBR Green Assays**		
*ONNV (−) strand detection*		
ONNV F S tag S	RT	**GGCCGTCATGGTGGCGAATAA** TACCACCAGGCGATCAAGGAGTC
ONNV R S	qPCR	aataaatcataaAACACTCGGTCGCATGGCTTCAAT
Tag S	qPCR	aataaatcataa**GGCCGTCATGGTGGCGAATAA**
*ONNV (+) strand detection*		
ONNV R S tag S	RT	**GGCCGTCATGGTGGCGAATAA** AACACTCGGTCGCATGGCTTCAAT
ONNV F S	qPCR	aataaatcataaTACCACCAGGCGATCAAGGAGTC
Tag S	qPCR	(see sequence above)
**TaqMan Assays**		
*ONNV (−) strand detection*		
ONNV F T tag T	RT	**GGCAGTATCGTGAATTCGATGC** ACGCGAGAAAACTTGCATCA
ONNV R T	qPCR	aataaatcataaTTTTTCCGGAGATGTTTTTATCTGT
Tag T	qPCR	aataaatcataa**GGCAGTATCGTGAATTCGATGC**
ONNV probe	qPCR	CCGCTGGAAAGGT
*ONNV (+) strand detection*		
ONNV R T tag T	RT	**GGCAGTATCGTGAATTCGATGC** TTTTTCCGGAGATGTTTTTATCTGT
ONNV F T	qPCR	aataaatcataaACGCGAGAAAACTTGCATCA
Tag T	qPCR	(see sequence above)
ONNV probe	qPCR	(see sequence above)
*CHIKV (−) strand detection*		
CHIKV F T tag T	RT	**GGCAGTATCGTGAATTCGATGC** GACGCAGAAACGCCCACATT
CHIKV R T	qPCR	aataaatcataaGTCCGCCCTTTGTCTACATGA
Tag T	qPCR	(see sequence above)
CHIKV probe	qPCR	TGCTTGCACACTGACGT
*CHIKV (+) strand detection*		
CHIKV R T tag T	RT	**GGCAGTATCGTGAATTCGATGC** GTCCGCCCTTTGTCTACATGA
CHIK F T	qPCR	aataaatcataaGACGCAGAAACGCCCACATT
Tag T	qPCR	(see sequence above)
CHIKV probe	qPCR	(see sequence above)

a The non-alphavirus tag sequences are shown in boldface.

b The AT-rich flap sequences (Afonina et al. 2007) are shown in lowercase.

### Strand specific quantitative Real-Time PCR

#### TaqMan® assays

To increase fluorescent signal strength during ssqPCR reactions, an AT-rich 12-nucleotide flap sequence (5′AATAAATCATAA 3′) was added to the 5′ end of tagged primers [18]. ssqPCR was performed with the appropriate combination of primers and TaqMan® (Applied Biosystems) probe (Table 1) using an ABI 7300 (Applied Biosystems). Each reaction contained 12.5 µl of 1X ABI Gene Expression Master Mix (Applied Biosystems), TaqMan® probe at a final concentration of 250 nM, forward and reverse primers, each at a final concentration of 900 nM, and 2 µl of diluted cDNA. Samples were run in triplicate. The standard cycling conditions were 50°C for 2 min, 95°C for 10 min, followed by 40 cycles of 95°C for 15 sec and 61°C for 1 min. Data collection occurred during the 61°C extension step.

#### SYBR® Green assays

ssqPCR was performed with the appropriate forward or reverse and tag-specific primer pair (Table 1). When a tag sequence was not present in the cDNA, ssqPCR was performed using only nsP1-specific forward and reverse primer pairs (Table 1). Each reaction contained 10 µl of 1X Power SYBR® Green Master Mix (Applied Biosystems), forward and reverse primers, each at a final concentration of 800 nM, and 2 µl of cDNA. The standard cycling conditions were 95°C for 10 min, followed by 40 cycles of 95°C for 15 sec, 56°C for 30 sec, 72°C for 30 sec, and to monitor potential non-specific amplification one cycle of 95°C for 15 sec, 60°C for 1 min, and 95°C for 15 sec. Data collection occurred during the 72°C extension step.

## Results

### Accurate quantification of specific viral RNA strands depends on the presence of a tag sequence in the cDNA

**Figure 1:**
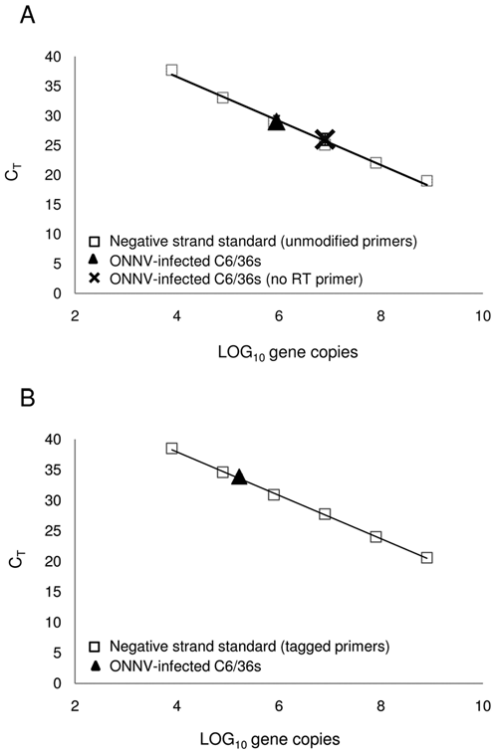
Detection of ONNV RNA with ssqPCR assays using unmodified or tagged primer systems. Quantification of ONNV (−) strand RNA with a ssqPCR assay using unmodified primers (A), or a tagged primer system (B) produces significantly different values (*P*<0.001). Amplification of cDNAs generated in an unprimed RT reaction with unmodified ONNV-specific primers (A).

The ability of ssqPCR assays that employ a tagged primer system to detect and quantify viral RNA of a specific polarity has been well demonstrated [8], [9], [11]. These assays incorporate a unique tag sequence into cDNA synthesized from a specific strand of viral RNA. A tag-specific primer used during PCR amplification ensures that only cDNA possessing the unique tag sequence is detected and quantified. Standard qPCR assays that employ unmodified primers have also been used to quantify specific strands of viral RNA [5], [6], [7], [10], [13], [16], but the accuracy of these measurements remains unclear. To directly compare the accuracy of commonly used qPCR strategies for measuring specific strands of viral RNA, total RNA was extracted from ONNV- infected mosquito cells and reverse transcribed using either an ONNV-specific forward primer possessing a unique 5′ tag sequence, or an unmodified ONNV-specific forward primer (Table 1). The respective cDNAs, with or without tag sequence, were qPCR amplified in the presence of SYBR® Green dye using a primer set containing a tag-specific forward primer and an ONNV-specific reverse primer, or with a primer set containing only ONNV-specific primers (Table 1). The amount of ONNV (−) strand RNA in the unknown sample was calculated from standard curves (Fig. 1; A and B). The standard curve generated for the assay using unmodified ONNV-specific primers had a slope of −3.4, coefficient of determination (*R^2^*) of 0.994, and amplification efficiency (Eff%) of 96.8%. The standard curve for the assay using a combination of tag-specific and ONNV-specific primers had a slope of −3.4, an *R^2^* of 0.999, and amplification efficiency of 96.8%. Both assays gave values for the quantity of ONNV (−) strand RNA that were within the acceptable ranges of their respective standard curves: 8.0×10^5^ copies of (−) strand RNA/µg of total RNA for the ssqPCR assay using unmodified primers, and 1.1×10^5^ copies of (−) strand RNA/µg of total RNA for the ssqPCR assay using a tagged primer system (Fig. 1; A and B). However, these values were found to be significantly different from each other (*P*<0.001; one-way ANOVA), suggesting that one or both of the ssqPCR assays was prone to error.

Accurate quantification of specific viral RNA strands with a ssqPCR assay depends on the assumption that the amount of cDNA quantified by qPCR closely approximates the amount of a specific strand of viral RNA in the RT reaction. As it has been demonstrated that cDNA can be transcribed from false-priming of RNA during an RT reaction in the absence of any specific primer [19], [20], [21], we hypothesized that the observed variation between the results obtained with our two assays was due to amplification of falsely-primed cDNAs during the qPCR step. The strand-specificity of ssqPCR assays using unmodified primers depends on the RT reaction, where only a single virus-specific primer is present. After the RT step, cDNA originating from either strand (through both specific and false priming) can be amplified by the virus-specific forward and reverse primers present during the qPCR step. Using a combination of virus-specific and tag-specific primers during qPCR is thought to limit amplification of dsDNA from falsely-primed cDNAs, as these molecules lack the unique tag sequences added during the RT step. Thus, ssqPCR assays using tag-specific primers are believed to better discern cDNAs transcribed from a specific strand of viral RNA from those transcribed from falsely-primed viral RNAs. To test this, an RT reaction was performed with the same total RNA (from ONNV-infected mosquito cells) used in the previously described experiments, but in the absence of any primer. The resultant cDNA was then used in qPCR reactions with the same primer sets used in the previously described experiments: a tag-specific forward primer and an ONNV-specific reverse primer, or unmodified ONNV-specific primers (Table 1). As expected, no amplification of falsely-primed cDNAs was detectable when the tag-specific and ONNV-specific primer were used together. However, a value of 7.4×10^6^ copies of ONNV RNA/µg of total RNA (the polarity of the RNA is unclear) was determined by standard curve when the two unmodified ONNV-specific primers were used together (Fig. 1A). These results suggest that the previously observed variation between our two ssqPCR assays was due to qPCR amplification of falsely-primed cDNAs by the unmodified virus-specific primers.

**Figure 2:**
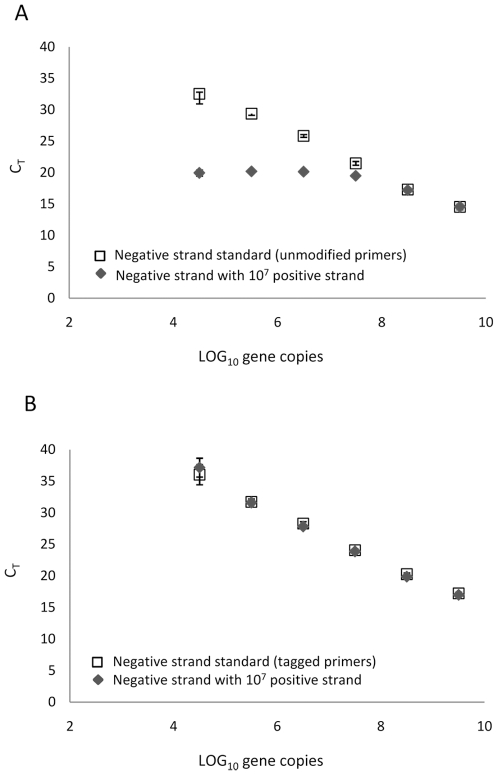
Strand specificity of ssqPCR assays using unmodified or tagged primer systems. Standard curves generated both in the presence or absence of a fixed amount of competing (+) strand cDNAs with a ssqPCR assay using unmodified primers (A) or with an assay using a tagged primer system (B).

To evaluate the accuracy of ssqPCR assays in the presence of cDNAs specifically transcribed from a competing viral RNA strand, we generated standard curves with our two ONNV (−) strand RNA ssqPCR assays in the presence or absence of a fixed amount of cDNA corresponding to ONNV (+) strand RNA. Standard curves produced with the unmodified primer set were very different depending on whether or not ONNV (+) strand cDNA was present during qPCR (Fig. 2A). In the absence of ONNV (+) strand cDNA, the standard curve had a slope of −3.6, an *R^2^* value of 0.990, and amplification efficiency of 89.6%. However, in the presence of ONNV (+) strand cDNA, the slope was −1.4, the *R^2^* value was 0.809, and the amplification efficiency was 417.9%. Only at dilutions in which ONNV (−) strand cDNA was present in excess of ONNV (+) strand cDNA were C_T_ values comparable between the two standard curves (Fig. 2A). Higher levels of cDNA from the competing (+) strands generally resulted in lower C_T_ values when compared with reactions that did not contain cDNA from the (+) strand, suggesting amplification of cDNA from both the intended target strand and the competing (+) strands. Standard curves generated with the tag-specific primer set were similar in the absence or presence of ONNV (+) strand cDNA: the slopes were −3.6 and −3.7, the *R^2^* values were 0.995 and 0.990, and amplification efficiencies were 89.6% and 86.3%, respectively (Fig. 2B). Overall, our results indicate higher relative levels of competing (+) strand cDNAs present during qPCR specifically inhibit accurate quantification of ONNV (−) strand RNA when unmodified virus-specific primers are used. However, accurate quantification of ONNV (−) strand RNA with a tag-specific primer set is unaffected by the presence of higher levels of ONNV (+) strand cDNA, as evidenced by the reproducibility of standard curves.

### DNA hydrolysis probes increase the sensitivity of qPCR with a tag-specific primer

SYBR® Green emits a strong fluorescent signal upon binding to dsDNA. Because the intensity of this fluorescent signal increases with the amount of dsDNA present, the dye can be used to detect and measure the accumulation of qPCR amplicons. However, amplification of nonspecific templates after many PCR cycles can result in additional fluorescence unrelated to any specific target. This nonspecific fluorescence has been shown to limit the sensitivity of dsDNA specific dyes for the detection and quantification of low-copy number targets [22], [23]. Because in alphavirus-infected cells (−) strand RNA synthesis ceases early in infection, concentrations of (−) strand RNA are likely to diminish with time [2]. Thus, SYBR® Green may not represent an ideal chemistry for detecting and measuring levels of alphavirus (−) strand RNA in infected cells, particularly later in infection when (−) strand RNA is less abundant. Previous work suggested DNA hydrolysis probes, which are sequence-specific, might provide increased sensitivity when used with ssqPCR assays [22]. In this case, the detection and measurement of amplification during qPCR is achieved by the fluorescent signal generated by a fluorophore released from a dual-labeled oligonucleotide probe. The fluorescent reporter dye is released from the 5′ end of the probe by the exonuclease activity of Taq polymerase, reducing proximity to a quencher dye at the 3′ end of the probe [24]. Thus in contrast to dsDNA dyes, no fluorescence is generated from amplification of nonspecific templates because the fluorescent signal is dependent on hydrolysis of the probe following hybridization to a specific target sequence.

**Figure 3:**
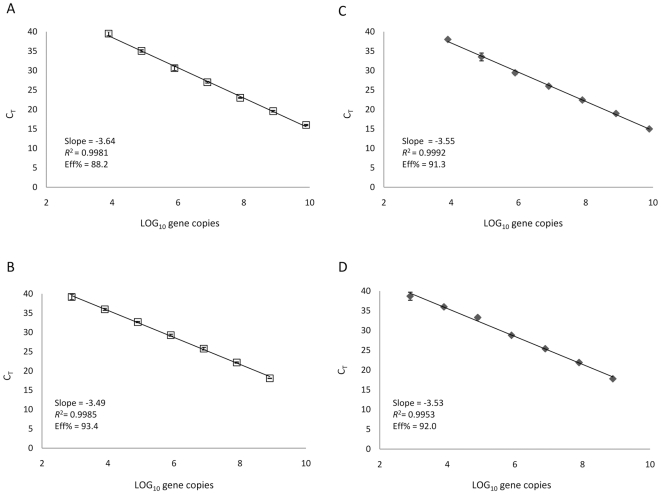
Sensitivity of ssqPCR assays using SYBR Green® or TaqMan®. Serial dilutions of ONNV (−) strand cDNAs quantified with tagged ssqPCR assays using SYBR Green® (A) or TaqMan® (B). Serial dilutions of ONNV (+) strand cDNAs quantified with tagged ssqPCR assays using SYBR Green® (C) or TaqMan® (D).

To determine if DNA hydrolysis probes increase the sensitivity of qPCR with a tag-specific primer, RNAs corresponding to ONNV (+) or (−) strands were synthesized in an in vitro transcription reaction. Following reverse transcription with a tagged forward or reverse primer (Table 1), ONNV (+) and (−) strand cDNAs were serially diluted and used in the generation of standard curves. Two standard curves were generated with each 10-fold dilution series of ONNV (+) or (−) strand cDNAs, one with SYBR® Green dye and the other with an ONNV-specific TaqMan® probe (Table 1). New tag-specific primer sets were designed for use with the TaqMan®-based detection strategy and chemistry (Table 1). The lowest dilution of either cDNA strand [(+) or (−)] that could be detected by qPCR with SYBR® Green contained 8×10^3^ copies of ONNV cDNA/reaction (Fig. 3; A and C). However, the lowest dilution detected with the TaqMan®-based assay contained only 8×10^2^ copies of ONNV cDNA/reaction (Fig. 3; B and D). These results indicate a greater dynamic range for assays using DNA hydrolysis probes in the quantification of low copy templates.

### Development and validation of ssqPCR assays for CHIKV

**Figure 4:**
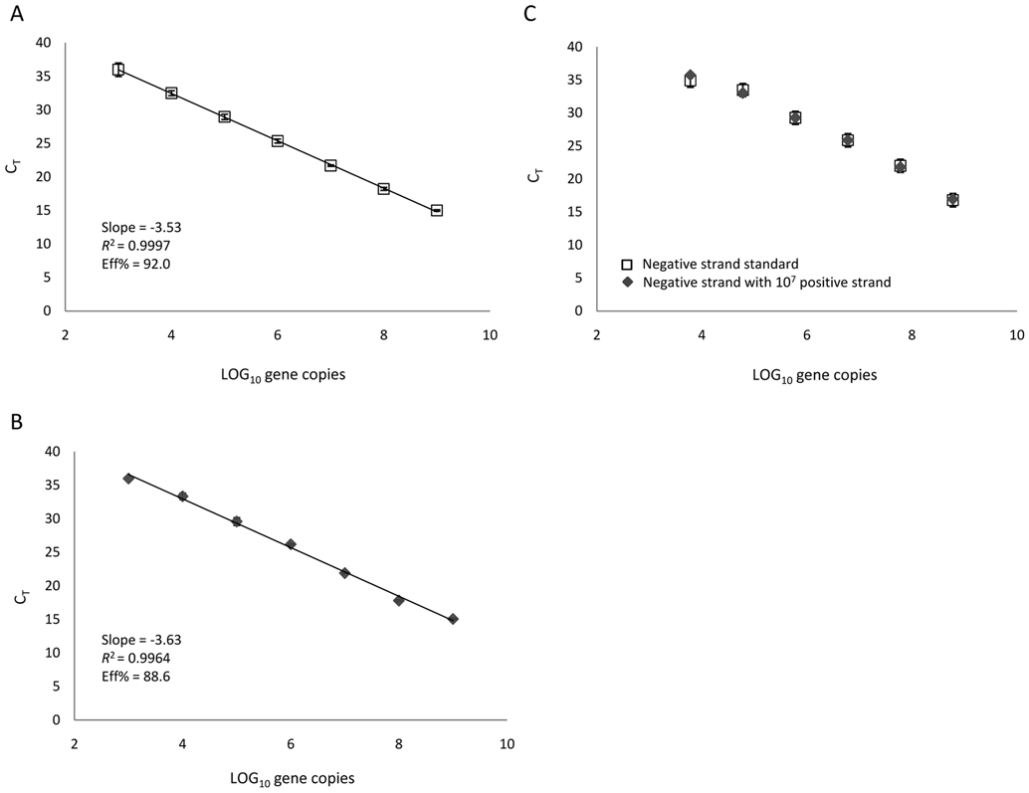
Strand specificity and sensitivity of CHIKV ssqPCR assays. Quantification of CHIKV cDNAs with a tagged ssqPCR (−) strand assay (A) or with a tagged ssqPCR (+) strand assay (B). Standard curves generated both in the presence or absence of a fixed amount of competing (+) strand cDNA with the tagged CHIKV ssqPCR (−) strand assay (C).

We next applied the information garnered in the previously described experiments to the design of ssqPCR assays for CHIKV, the cause of recent large scale outbreaks of debilitating disease in India and islands in the Indian Ocean [25], [26]. Primer sets and TaqMan® probe sequences are listed in Table 1. Standard curves were generated and are shown in figure 4 (A and B). The lowest 10-fold dilution of either cDNA strand [(+) or (−)] that could be detected with the CHIKV ssqPCR assays contained 1×10^3^ copies of CHIKV cDNA/reaction (Fig. 4; A and B). Reactions containing 100 copies of either cDNA strand did not consistently generate threshold crossing fluorescence in less than 40 cycles, indicating that this template concentration was outside the dynamic range of the assays. The specificity of the (−) strand assay was unaffected by the presence of cDNAs transcribed from competing (+) strand viral RNAs. Standard curves generated with the primer set containing the tag-specific primer had a slope of −3.3,an *R^2^* value of 0.987, and amplification efficiency of 100.9% in the absence of CHIKV (+) strand cDNA, and a slope of −3.5,an *R^2^* value of 0.973, and amplification efficiency of 93.07% in the presence of CHIKV (+) strand cDNA (Fig. 4C). As an additional test of the strand specificity of our CHIKV assays, cDNA dilutions used in the generation of standard curves were used in qPCR reactions with the primer set for the opposite strand. Amplification of (−) strand cDNA was undetectable with the (+) strand primer set, and conversely amplification of (+) strand cDNA was undetectable with the (−) strand primer set (data not shown), confirming a high level of fidelity for the intended target strand.

## Discussion

Because RNA virus genomes must be efficiently copied in an infected cell, ssqPCR assays are valuable tools for the detection and quantification of replicating virus. However, the amount of cDNA quantified during qPCR must accurately reflect amounts of a specific viral RNA strand in the RT reaction. To determine how best to accomplish this, we developed several different ssqPCR assays for ONNV and then compared various parameters of those assays with each other.

We have shown that accurate quantification of ONNV (−) strand RNA is inhibited by the presence of relatively higher levels of cDNA generated from the competing (+) strand RNA, when standard unmodified ONNV-specific primers are used for reverse transcription and qPCR (Fig. 2B). Falsely-primed cDNAs have previously been demonstrated following reverse transcription of viral RNAs from dengue virus-infected cells and in vitro transcribed dengue and hepatitis C virus RNAs in the absence of any specific primer [20], [21]. Several mechanisms have been proposed to explain how false-priming may occur during the RT step, including self-priming of the reverse transcriptase from secondary hairpin structures present in highly folded viral RNA, and random-priming by short endogenous or exogenous nucleic acids [19], [20], [21]. Regardless of mechanism, we also demonstrated false priming of viral RNAs during reverse transcription of RNA extracted from ONNV infected cells (Fig. 1A). Although it was not possible to quantify the amount of falsely-primed cDNA that was generated from a specific strand of viral RNA, it is reasonable to presume that ratios of falsely-primed products approximate ratios of (+) and (−) strand RNAs in the infected cell. Because imbalances in the synthesis of genomic RNAs and their full-length complements are common during RNA virus infections, false priming of the more abundant strand during the RT step of the assay is likely to inhibit accurate quantification of the less abundant strand, when standard unmodified virus-specific primers are used for reverse transcription and qPCR. However, we have shown that the inhibitory effects of falsely-primed cDNAs on the accuracy of ssqPCR assays can be eliminated. When a unique tag sequence is added to cDNAs generated from ONNV (−) strands during reverse transcription, accurate quantification of ONNV (−) strand RNA is possible with a tag-specific primer, even in the presence of higher levels of ONNV (+) strand cDNA (Fig. 2B). Amplification of falsely-primed cDNAs generated from the competing (+) strand RNA are also undetectable during qPCR, as the tag-specific primer cannot anneal to falsely-primed products lacking a complementary sequence (Fig. 1; A and B).

The sensitivity of our ONNV (−) strand ssqPCR tag-specific assays was determined with serial 10-fold dilutions of cDNA generated from in vitro transcribed ONNV RNAs. With our SYBR® Green assay we were able to detect 8000 copies per reaction but could not detect 800 copies per reaction, indicating a lower limit of detection somewhere within this range. However, the limit of low-copy number detection decreased to somewhere between 800 (which could be detected) and 80 (which could not be detected) copies per reaction when a TaqMan® DNA hydrolysis probe was used to monitor amplification. The increased sensitivity likely resulted from eliminating the non-specific amplification of dsDNA products that is common when monitoring the generic fluorescence emitted by dsDNA-binding dyes. The sensitivities of our ONNV (+) strand ssqPCR tag-specific assays were determined to be identical to those of the (−) strand assays, with both detection strategies and chemistries. In the case of alphaviruses, the increased sensitivity of assays incorporating DNA hydrolysis probes should be useful, particularly when quantifying much less abundant (−) strands.

Finally, using the information obtained by directly comparing various parameters of multiple ONNV-specific ssqPCR assays, we developed and validated assays to detect and quantify CHIKV (−) and (+) strand RNAs. Serial dilutions of cDNA generated from in vitro transcribed CHIKV RNAs were used to confirm strand-specificity and determine sensitivity. Amplification of dsDNA was undetectable when the (+) strand primer set was used with cDNA derived from the (−) strand at all concentrations tested. The reverse was also found to be true using the (−) strand primer set and cDNA generated from (+) strand RNA. The accuracy of the CHIKV (−) strand assay was confirmed in the presence of cDNAs transcribed from competing (+) strand RNAs (Fig. 4C). The sensitivity of our CHIKV ssqPCR assays was determined to be between 1000 and 100 copies per reaction.

In summary, we have developed and validated two new ssqPCR assays for the medically important alphaviruses, CHIKV and ONNV. These assays will be useful in studies to determine how persistent alphavirus infections are maintained in the vector host, and in the detection and quantification of replicating virus from clinical specimens and potential reservoir hosts. In the course of developing these assays, we have shown that accurate quantification of cDNAs generated from specific strands of viral RNA, in the presence of higher levels of falsely-primed cDNA products generated from competing RNA strands, requires incorporation of a unique tag sequence during reverse transcription, which in combination with a tag-specific primer can be used to specifically amplify cDNAs corresponding to the intended target strand during qPCR. While it was also possible to quantify specific strands of viral RNA with assays employing unmodified virus-specific primers, the accuracy of these measurements depends on conditions in which lower relative levels of cDNA generated from the competing strand are present during qPCR. Therefore, previously reported results obtained with assays of this type should be interpreted with caution, particularly when the assay in question has been used to measure amounts of anti-genomic strands, which are typically less abundant than genomic RNAs in cells and tissues infected with RNA viruses.

## References

[1] Strauss JH, Strauss EG (1994). The alphaviruses: gene expression, replication, and evolution.. Microbiol Rev.

[2] Sawicki DL, Sawicki SG (1980). Short-lived minus-strand polymerase for Semliki Forest virus.. J Virol.

[3] Kim KH, Rumenapf T, Strauss EG, Strauss JH (2004). Regulation of Semliki Forest virus RNA replication: a model for the control of alphavirus pathogenesis in invertebrate hosts.. Virology.

[4] Shirako Y, Strauss JH (1994). Regulation of Sindbis virus RNA replication: uncleaved P123 and nsP4 function in minus-strand RNA synthesis, whereas cleaved products from P123 are required for efficient plus-strand RNA synthesis.. J Virol.

[5] Yuki N, Matsumoto S, Tadokoro K, Mochizuki K, Kato M (2006). Significance of liver negative-strand HCV RNA quantitation in chronic hepatitis C.. J Hepatol.

[6] Wang WK, Sung TL, Tsai YC, Kao CL, Chang SM (2002). Detection of dengue virus replication in peripheral blood mononuclear cells from dengue virus type 2-infected patients by a reverse transcription-real-time PCR assay.. J Clin Microbiol.

[7] Richardson J, Molina-Cruz A, Salazar MI, Black Wt (2006). Quantitative analysis of dengue-2 virus RNA during the extrinsic incubation period in individual Aedes aegypti.. Am J Trop Med Hyg.

[8] Purcell MK, Hart SA, Kurath G, Winton JR (2006). Strand-specific, real-time RT-PCR assays for quantification of genomic and positive-sense RNAs of the fish rhabdovirus, Infectious hematopoietic necrosis virus.. J Virol Methods.

[9] Komurian-Pradel F, Perret M, Deiman B, Sodoyer M, Lotteau V (2004). Strand specific quantitative real-time PCR to study replication of hepatitis C virus genome.. J Virol Methods.

[10] Hashimoto Y, Valles SM (2008). Detection and quantitation of Solenopsis invicta virus-2 genomic and intermediary replicating viral RNA in fire ant workers and larvae.. J Invertebr Pathol.

[11] Gu C, Zheng C, Shi L, Zhang Q, Li Y (2007). Plus- and minus-stranded foot-and-mouth disease virus RNA quantified simultaneously using a novel real-time RT-PCR.. Virus Genes.

[12] Castillo I, Rodriguez-Inigo E, Lopez-Alcorocho JM, Pardo M, Bartolome J (2006). Hepatitis C virus replicates in the liver of patients who have a sustained response to antiviral treatment.. Clin Infect Dis.

[13] Campbell CL, Keene KM, Brackney DE, Olson KE, Blair CD (2008). Aedes aegypti uses RNA interference in defense against Sindbis virus infection.. BMC Microbiol.

[14] Bartolome J, Lopez-Alcorocho JM, Castillo I, Rodriguez-Inigo E, Quiroga JA (2007). Ultracentrifugation of serum samples allows detection of hepatitis C virus RNA in patients with occult hepatitis C.. J Virol.

[15] Anwar A, August JT, Too HP (2006). A stem-loop-mediated reverse transcription real-time PCR for the selective detection and quantification of the replicative strand of an RNA virus.. Anal Biochem.

[16] Anderson JR, Rico-Hesse R (2006). Aedes aegypti vectorial capacity is determined by the infecting genotype of dengue virus.. Am J Trop Med Hyg.

[17] Lin L, Fevery J, Hiem Yap S (2002). A novel strand-specific RT-PCR for detection of hepatitis C virus negative-strand RNA (replicative intermediate): evidence of absence or very low level of HCV replication in peripheral blood mononuclear cells.. J Virol Methods.

[18] Afonina I, Ankoudinova I, Mills A, Lokhov S, Huynh P (2007). Primers with 5′ flaps improve real-time PCR.. Biotechniques.

[19] Timofeeva AV, Skrypina NA (2001). Background activity of reverse transcriptases.. Biotechniques.

[20] Peyrefitte CN, Pastorino B, Bessaud M, Tolou HJ, Couissinier-Paris P (2003). Evidence for in vitro falsely-primed cDNAs that prevent specific detection of virus negative strand RNAs in dengue-infected cells: improvement by tagged RT-PCR.. J Virol Methods.

[21] Gunji T, Kato N, Hijikata M, Hayashi K, Saitoh S (1994). Specific detection of positive and negative stranded hepatitis C viral RNA using chemical RNA modification.. Arch Virol.

[22] Wittwer CT, Herrmann MG, Moss AA, Rasmussen RP (1997). Continuous fluorescence monitoring of rapid cycle DNA amplification.. Biotechniques.

[23] Higuchi R, Fockler C, Dollinger G, Watson R (1993). Kinetic PCR analysis: real-time monitoring of DNA amplification reactions.. Biotechnology (N Y).

[24] Holland PM, Abramson RD, Watson R, Gelfand DH (1991). Detection of specific polymerase chain reaction product by utilizing the 5′----3′ exonuclease activity of Thermus aquaticus DNA polymerase.. Proc Natl Acad Sci U S A.

[25] Powers AM, Logue CH (2007). Changing patterns of chikungunya virus: re-emergence of a zoonotic arbovirus.. J Gen Virol.

[26] Enserink M (2008). Entomology. A mosquito goes global.. Science.

